# Early Biological Therapy in Operated Crohn’s Disease Patients Is Associated With a Lower Rate of Endoscopic Recurrence and Improved Long-term Outcomes: A Single-center Experience

**DOI:** 10.1093/ibd/izac110

**Published:** 2022-05-28

**Authors:** Ferdinando D’Amico, Olga Tasopoulou, Gionata Fiorino, Alessandra Zilli, Federica Furfaro, Mariangela Allocca, Pierpaolo Sileri, Antonino Spinelli, Laurent Peyrin-Biroulet, Silvio Danese

**Affiliations:** Gastroenterology and Endoscopy, IRCCS Ospedale San Raffaele and Vita-Salute San Raffaele University, Milan, Italy; Department of Biomedical Sciences, Humanitas University, Pieve Emanuele, Milan, Italy; Department of Biomedical Sciences, Humanitas University, Pieve Emanuele, Milan, Italy; Gastroenterology and Endoscopy, IRCCS Ospedale San Raffaele and Vita-Salute San Raffaele University, Milan, Italy; Gastroenterology and Endoscopy, IRCCS Ospedale San Raffaele and Vita-Salute San Raffaele University, Milan, Italy; Gastroenterology and Endoscopy, IRCCS Ospedale San Raffaele and Vita-Salute San Raffaele University, Milan, Italy; Gastroenterology and Endoscopy, IRCCS Ospedale San Raffaele and Vita-Salute San Raffaele University, Milan, Italy; Gastrointestinal Surgery Unit, IRCCS Ospedale San Raffaele, MilanItaly; Department of Biomedical Sciences, Humanitas University, Pieve Emanuele, Milan, Italy; Division of Colon and Rectal Surgery, IRCCS Humanitas Research Hospital, Rozzano, Milan, Italy; University of Lorraine, CHRU-Nancy, Department of Gastroenterology, F-54000 Nancy, France; University of Lorraine, Inserm, NGERE, F-54000 Nancy, France; Gastroenterology and Endoscopy, IRCCS Ospedale San Raffaele and Vita-Salute San Raffaele University, Milan, Italy

**Keywords:** Crohn’s disease, surgery, inflammatory bowel disease, biological therapy

## Abstract

**Background:**

Two-thirds of Crohn’s disease (CD) patients require surgery during their disease course. However, surgery is not curative, and endoscopic recurrence is observed in up to 90% of cases. Our aim was to investigate the impact of postoperative biological therapy on the incidence of endoscopic recurrence and long-term outcomes in CD patients.

**Methods:**

This retrospective cohort study was conducted at the Humanitas Research Hospital–IRCCS (Milan, Italy) between 2014 and 2021. All consecutive CD patients who underwent surgery and colonoscopy at 6-12 months postoperatively were eligible for inclusion.

**Results:**

A total of 141 patients were included (42.6% female, mean age 44 years). Median follow-up was 28 months. About one-third of patients were treated with biologics at baseline colonoscopy. A higher rate of endoscopic recurrence was detected in patients without biologic therapy at the time of colonoscopy compared with those treated (80.8% vs 45.2%, *P < .*0001). Hospitalization and surgery occurred more in untreated patients than in subjects undergoing biological therapy (12.1% vs 0.0%, *P = .*01). The Kaplan-Meier curves showed that the no treatment group at baseline had a >23.3% 5-year rate of hospitalization and surgery (log-rank *P = .*0221) and a >49.7% 5-year rate of medical therapy escalation (log-rank *P = .*0013) compared with the treatment arm. In the logistic regression model, absence of biologic therapy was independently associated with the risk of endoscopic disease recurrence (odds ratio, 0.22; 95% CI, 0.1-0.51; *P = .*0004).

**Conclusion:**

Operated CD patients treated early with biologics experience decreased rates of endoscopic recurrence and improved long-term outcomes.

**Key Messages**

**What is already known?**

Patients undergoing Crohn’s disease–related surgery are treated with biological therapy in case of risk factors for recurrence or if there is an endoscopic recurrence of disease.

**What is new here?**

Crohn’s disease patients treated with biological therapy postoperatively experience a reduced rate of hospitalization and surgery regardless of endoscopic disease recurrence and risk factors.

**How can this study help patient care?**

Early treatment with postoperative biological therapy may prevent disease recurrence and improve long-term outcomes of patients with Crohn’s disease.

## Introduction

Crohn’s disease (CD) is a chronic and disabling inflammatory disorder with a relapsing and remitting course.^1,2^ The introduction of biologic therapies such as anti-tumor necrosis factor alpha (TNF-α), vedolizumab, and ustekinumab has revolutionized the management of CD patients, significantly improving their prognosis and quality of life.^3–6^ However, due to multiple complications (ie, strictures, abscesses, fistulas) and failure of medical therapy, two-thirds of patients undergo surgery during their lifetime.^7^ Unfortunately, surgery is not curative and endoscopic disease recurrence occurs in up to 90% of patients at 3 years, and as many as 40% undergo an additional surgery within 10 years.^1,2,8^ To date, operated patients are monitored endoscopically 6-12 months after surgery, and biologic therapy is started in case of endoscopic recurrence (Rutgeerts score ≥2).^9^ Strictures, fistulas, active tobacco use, prior intestinal resection (especially >50 cm), early age of disease onset, and perianal disease have been associated with an increased rate of endoscopic relapse.^10^ For this reason, biologic therapy is started immediately after surgery if these risk factors are present in order to prevent postoperative relapse.^2,11,12^ Importantly, patients without risk factors who do not experience disease activity at postoperative endoscopic control are not treated with biologic therapy and do not initiate maintenance therapy.^9,10,13^ So far, there are no data on the long-term outcomes of these patients, and it is not known whether postoperative treatment with biologics may have an impact in reducing the risk of relapse. Growing evidence reveals that there is a window of opportunity for the management of CD patients to prevent disease progression and complications.^14^ Indeed, early treatment with biologic therapy has been associated with better disease control and prognosis.^15^ However, there are no data on the role of early use of biological agents in operated CD patients, and their treatment remains challenging for clinicians. The aim of this study was to investigate the impact of postoperative biologic therapy on the incidence of endoscopic relapse and long-term negative outcomes in CD patients, stratifying the results based on whether they were receiving biological drugs at baseline colonoscopy.

## Methods

### Study Design and Inclusion Criteria

This study was an observational retrospective cohort study. Patients were selected from the electronic medical records of the IBD center of the Humanitas Research Hospital—IRCCS (Rozzano, Milan, Italy), spanning from January 2014 to May 2021. All adult patients with a confirmed diagnosis of CD for at least 3 months undergoing at least one CD–related surgery were eligible for inclusion. All resections at the time of baseline were curative. Additionally, only patients with at least 1 colonoscopy at 6-12 months after surgery and available clinical data within 1 month from the endoscopic examination were included in the study. When available, all other data were collected, including biopsies with histological reports, radiological examinations (both small bowel ultrasound and magnetic resonance [MR] enterography), and fecal calprotectin values. In daily clinical practice, imaging and biopsy specimen collection are not regularly performed and thus were not available in all patients. The first available colonoscopy was considered as baseline, and patients were monitored over time to evaluate both the recurrence of disease postoperatively and the onset of negative disease outcomes. If a patient underwent multiple colonoscopies, all available data were collected; and the association between changes in disease endoscopic activity and occurrence of negative outcomes was evaluated. The results were then stratified based on whether the patients were under treatment with biologic agents or not at the baseline endoscopy. All patients under the age of 18 at time of enrollment and patients with ulcerative colitis or unclassified colitis were excluded.

### Data Collection

The clinical, biochemical, endoscopic, histologic, and radiologic data were extracted directly from the patient’s electronic medical records from the Humanitas Research Hospital. For each patient, demographic data including, gender, age, date of birth, date of diagnosis, and age at diagnosis were collected. In addition, smoking status, disease extent, presence of upper CD, perianal disease, family history of IBD, and concomitant rheumatologic disease were evaluated. All preoperative IBD medical therapies were reported, specifically corticosteroids (oral or systemic), immunomodulators (thiopurines, methotrexate), TNF-α inhibitors (infliximab, adalimumab, certolizumab), anti-integrins (vedolizumab), interleukin 12/23 inhibitors (ustekinumab), taking into consideration both the start and the end date of each medication and the reason for drug discontinuation. Clinical activity was assessed using the Harvey-Bradshaw Index (HBI), and disease activity was defined as HBI ≥6. The HBI was invariably recorded in the electronic medical records during every clinical visit. Biochemical disease activity was defined as fecal calprotectin >100 μg/g. Endoscopical activity was measured using the Rutgeerts score, and a score ≥i2 was considered to be disease relapse. Histologic disease activity was based on the presence or absence of neutrophils at the level of the epithelium. Finally, a bowel thickness *>*3 mm was adopted to identify radiologic disease activity on MR enterography or small bowel ultrasound. All postoperative biologic treatments were also investigated, and negative disease outcomes at the latest follow-up were reported: escalation of medical therapy (ie, initiation of any biologic drug, dose or interval therapy optimization, switch or swap to another drug class), CD-related hospitalization (≥3 days), need for CD-related surgery, development of colorectal dysplasia or malignancy, and death from all causes.

### Statistical Analysis

Descriptive statistics was presented as mean ± standard deviation (SD) or median in the case of continuous variables. Categorical variables were described as percentages. A binary logistic regression model was used to investigate the association between patients’ characteristics at baseline and long-term negative outcomes. The proportional hazard assumption was tested by statistical and graphical diagnostics based on the scaled Schoenfeld residuals. The log-linearity assumption was performed by introducing in the model the squared covariable and using a likelihood ratio statistic test. A Kaplan-Meier survival curve was also produced to illustrate the rate of the selected negative outcomes as a function of time. Any *P* values less than 0.05 were considered statistically significant. All statistical tests were 2-sided. Stata software was used for statistical analyses (Stata Corp., College Station, TX, USA).

## Results

### Patients’ Characteristics

Table 1 summarizes the patients’ characteristics at the time of their surveillance colonoscopy. A total of 141 patients were included (42.6% female), with a mean age of 45.1 ± 14.2 years. According to the Montreal Classification, most patients were diagnosed between the age of 17 and 40 years old (*n* = 87, 61.7%). About two-thirds of patients had ileal disease at diagnosis (*n* = 88, 62.4%). The stricturing phenotype was the most frequent at diagnosis (*n* = 77, 54.6%). Perianal disease was detected in about one-fifth of patients (*n* = 24, 17.0%), whereas upper GI involvement occurred in few patients (*n* = 5, 3.5%). The majority of subjects were former smokers (*n* = 61, 43.2%) or never smokers (*n* = 52, 36.9%). The most frequently used preoperative drugs were systemic (*n* = 66, 46.8%) and topical steroids (*n* = 45, 31.9%). Concerning biologic therapy, the most used agents were adalimumab (*n* = 31, 22.0%), infliximab (*n* = 13, 9.2%), ustekinumab (*n* = 4, 2.8%), and vedolizumab (*n* = 3, 2.1%). About one-third of the patients were under treatment at baseline colonoscopy (*n* = 42, 29.8%). The median duration of biologic therapy at the time of index colonoscopy was 9.8 months (range 5-12, Table 2). The indications for treatment were stricturing phenotype (*n* = 20, 48%), penetrating phenotype (*n* = 15, 36%), active smoking (*n* = 10, 24%), multiple surgeries (*n* = 7, 17%), and young age of onset (*n* = 3, 7%). The median duration of biologic therapy for this subgroup was 19 months (range 7-87).

**Table 1.** Patients’ characteristics at baseline.

	N	%/mean	SD	Median
Age	141	45.1	14.2	45.0
Age at Diagnosis	141	33.9	12.8	32.0
**Sex**				
Male	81	57.4		
Female	60	42.6		
**Smoking Status**				
Non smoker	52	36.9		
Former smoker	61	43.2		
Active smoker	28	19.9		
**Montreal Classification**				
**Age**				
A1 <16	8	5.7		
A2 (17-40)	87	61.7		
A3 (>40)	28	32.6		
**Location**				
L1: ileal	88	62.4		
L2: colonic	3	2.1		
L3: ileocolonic	50	35.5		
L4: isolated upper GI	0	0.0		
**Behavior**				
B1: nonstricturing, nonpenetrating	19	13.5		
B2: stricturing	77	54.6		
B3: penetrating	45	31.9		
**Preoperative History**				
Topical steroids	45	31.9		
Systemic steroids	66	46.8		
Thiopurine	38	27.0		
Methotrexate	6	4.3		
Infliximab	13	9.2		
Adalimumab	31	22.0		
Vedolizumab	3	2.1		
Ustekinumab	4	2.8		
**Familiarity for IBD**				
Positive	17	12.1		
**Perianal Disease**	24	17.0		
**Upper GI Involvement**	5	3.5		
**Concomitant Rheumatologic Disease**				
None	131	93.0		
Spondylarthritis	2	1.4		
Enteropathic arthritis	2	1.4		
Ankylosing spondylitis	2	1.4		
Sacroileitis	4	2.8		

Abbreviations: SD, standard deviation, IBD, inflammatory bowel disease, GI, gastrointestinal.

**Table 2.** Patients’ characteristics stratified according to biologic therapy at baseline.

	N	Total N = 141	SD	N	Biologic Therapy at Baseline N = 42 (29.8%)	SD	N	No Biologic Therapy at Baseline N = 99 (70.2%)	SD	*P* ^a^
		%/mean			%/mean			%/mean		
Age	141	45.1	14.2	42	44.0	13.7	99	45.6	14.5	0.5120
Age at diagnosis	141	33.9	12.8	42	31.7	12.8	99	34.9	12.5	0.1698
**Gender**										0.2847
Male	81	57.4		27	64.3		54	54.5		
Female	60	42.6		15	35.7		45	45.5		
**Smoking Status**										0.0852
Non smoker	52	36.9		20	47.6		32	32.3		
Ex smoker	61	43.2		12	28.6		49	49.5		
Smoker	28	19.9		10	23.8		18	18.2		
**Montreal Classification**										
**Age**										0.1788
A1 < 16	8	36.9		3	7.2		5	5.0		
A2 (17-40)	87	43.3		30	71.4		57	57.6		
A3 (>40)	28	19.9		9	21.4		37	37.4		
**Location**										0.5418
L1: ileal	88	62.4		25	59.5		63	63.6		
L2: colonic	3	2.1		0	0.0		3	3.0		
L3: ileocolonic	50	35.5		17	40.5		33	33.3		
L4: isolated upper	0	0.0		0	0.0		0	0.0		
GI										
**Behavior**										0.5331
B1: nonstricturing,	19	13.5		7	16.7		12	12.1		
nonpenetrating										
B2: stricturing	77	54.6		20	47.6		57	57.6		
B3: penetrating	45	31.9		15	35.7		30	30.3		
**Preoperative History**										
Topical steroids	45	31.9		13	31.0		32	32.3		0.8731
Systemic steroids	66	46.8		24	57.1		42	42.4		0.1092
Thiopurine	38	27.0		16	38.1		22	22.2		0.0521
Methotrexate	6	4.3		3	7.1		3	3.0		0.2685
**Familiarity for IBD**										0.5474
Positive	17	12.1		4	9.5		13	13.1		
Negative	124	87.9		38	90.5		86	86.9		
**Perianal Disease**										0.0008
Present	24	17.0		14	33.3		10	10.1		
Absent	117	83.0		28	66.7		89	89.9		
**Upper GI**										0.6261
Present	5	3.5		1	2.4		4	4.0		
Absent	136	96.5		41	97.6		95	96.0		
**Concomitant Rheumatologic disease**										0.0829
Present	10	7.1		5	11.9		5	94.9		
Absent	131	92.9		31	88.1		94	5.1		

Abbreviations: SD, standard deviation, IBD, inflammatory bowel disease, GI, gastrointestinal,.

^a^The χ^2^ test for qualitative variables and Student *t* test for quantitative variables.

### Disease Relapse and Long-term Outcomes

Median follow-up was 28 months (range 11-110). Ninety-nine (70.2%) patients experienced endoscopic disease relapse at the baseline colonoscopy, whereas 7 patients (6.8%) presented histologic disease relapse. Regarding long-term negative outcomes, 3 patients (2.1%) underwent surgery (1 right hemicolectomy, 1 ileocolic resection, and 1 total colectomy), and 9 patients (6.4%) were hospitalized during the follow-up, with a mean hospitalization duration of 7.38 ± 11.39 days. About half of the patients (*n* = 71 of 141, 50.4%) experienced 1 or more strategies of medical therapy escalation, including initiation of biologic therapy (*n* = 71 of 141, 50.4%), dose (*n* = 5, 5%) or interval (*n* = 30, 21%) optimization, and switch (*n* = 18, 13%) or swap (*n* = 1, 1%) to another biologic agent. There was no incidence of colorectal dysplasia or cancer. One death (*n* = 1, 1%) due to a complicated nosocomial infection occurred in the arm not undergoing biologic therapy.

### Disease Relapse Stratified by Biologic Therapy

Patients not receiving biologic therapy at baseline experienced a higher rate of endoscopic disease relapse compared with those receiving early postoperative therapy with biological agents (80.8% vs 45.2%, *P = .*000024). Similarly, histologic disease activity at baseline colonoscopy was identified only among patients not treated with biologics (10.3% vs 0.0%, *P = .*04928; Table 3).

**Table 3.** Endoscopic and histologic disease relapse based on biologic therapy.

Colonoscopy at 6-12 months postoperatively	Total N = 141		Biologic Therapy at Baseline N = 42 (29.8%)		No Biologic Therapy at Baseline N = 99 (70.2%)		*P* ^a^
	N	%	N	%	N	%	
Endoscopic relapse	99	70.2	19	45.2	80	80.8	<0.0001
Endoscopic remission	42	29.8	23	54.8	19	19.2	
Histologic activity	7	6.8	0	0.0	7	10.3	0.04928
Histologic remission	96	89.7	42	100.0	61	89.7	

### Long-term Outcomes Stratified by Biologic Therapy 

**Figure 1.**
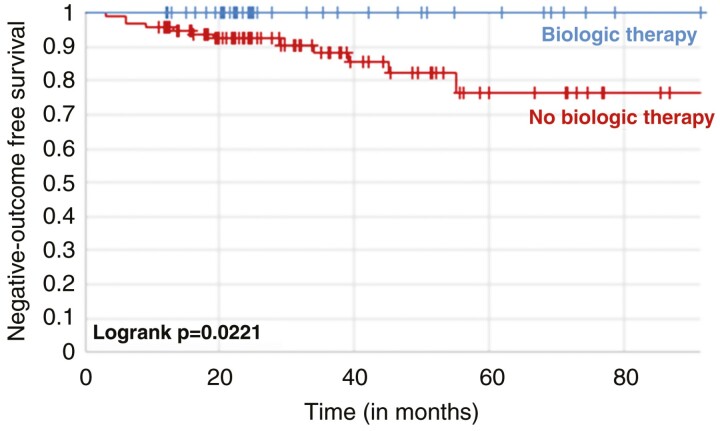
Kaplan-Meier survival curves showing the risk of hospitalization and surgery in patients with Crohn’s disease stratified by biologic therapy or not at baseline.

**Figure 2.**
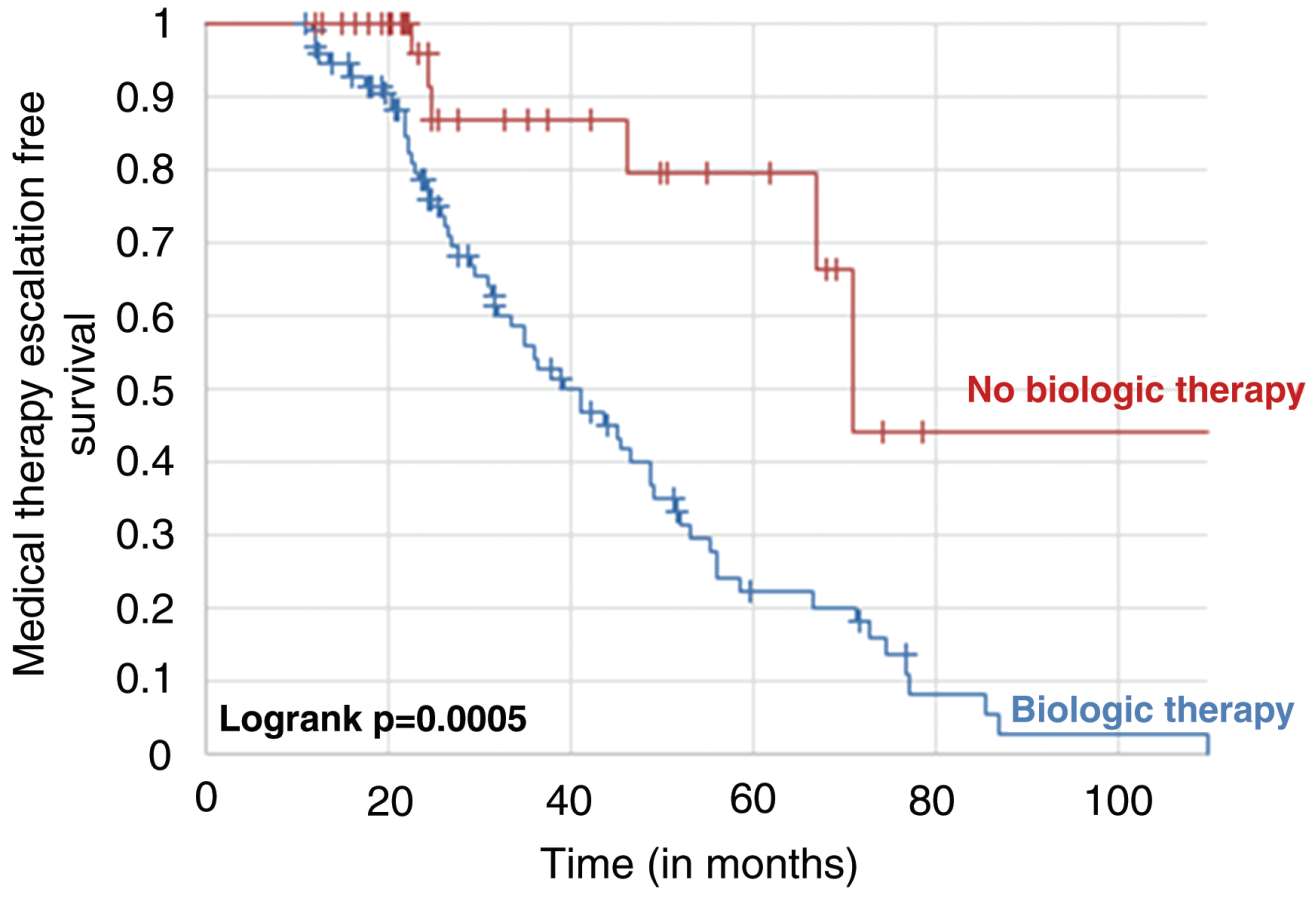
Kaplan-Meier survival curves showing the risk of medical therapy escalation in patients in patients with Crohn’s disease stratified by biologic therapy or not at baseline.

Overall, 11 patients of the no-treatment group at baseline experienced hospitalization or surgery during follow-up. In comparison, no patient treated with biological drugs at baseline was operated or hospitalized (11.1% vs 0.0%, *P = .*01833). In the Kaplan-Meier survival curve (Figure 1), patients not undergoing biological therapy at baseline colonoscopy had a 23.3% higher risk of experiencing hospitalization or surgery at 5 years compared with those treated (log rank *P = .*02221). Moreover, the rate of medical therapy escalation was significantly lower in the treatment group compared with the no-treatment group (14.0% vs 66.0%, *P < .*00001). The Kaplan-Meier survival curve (Figure 2) confirmed that patients not being treated at baseline colonoscopy had a 57.4% higher risk of experiencing medical therapy escalation at 5 years (log rank *P = .*005).

### Predictors of Outcomes

Biologic therapy at baseline colonoscopy was identified as a protective factor against endoscopic relapse (odds ratio [OR], 0.22; 95% CI, 0.1-0.51; *P = .*0004; Table 4). However, history of multiple surgeries was associated with a higher risk for surgery and hospitalization (OR, 5.19; 95% CI, 1.40-19.26; *P = .*013; Table 5). History of smoking or being a current smoker were risk factors for medical therapy escalation (OR, 2.51 95% CI, 1.06-4.35; *P = .*033; Table 6). The rate of negative outcomes was not affected by the type of biologic agent.

**Table 4.** Factors associated with endoscopic relapse

	N	Endoscopic Relapse		Logistic regression			
		n	%	Odds Ratio	95% CI		*P*
					Lower	Upper	
**Biologic therapy at baseline colonoscopy**							0.0004
No	99	80	80.1				
Yes	42	19	45.2	0.22	0.1-0.51		
**Multiple Surgeries**							0.4062
No	121	84	69.4				
Yes	20	15	75.0	1.68	0.49-5.76		
**Smoking History**							
Nonsmoker	52	32	61.5				
Smoker/Ex-smoker	89	67	75.2	1.57	0.70-3.51		0.2694
**Perianal Disease**							0.3838
Absent	117	67	57.3				
Present	24	13	54.1	0.63	0.23-1.77		
**Fistulizing Disease**							0.2241
Absent	96	71	74.0				
Present	45	28	62.2	0.60	0.27-1.37		

**Table 5.** Factors associated with surgery and hospitalization.

	N	Surgery and Hospitalization		Logistic Regression			
		*n*	% Odds ratio		95% CI		*P*
					lower	upper	
**Multiple Surgeries**							0.0139
No	121	5	4.1				
Yes	20	7	35.0	5.19	1.40-19.26		
**Smoking History**							0.2095
Non smoker	52	2	3.8				
Smoker/Former smoker	89	10	11.2	2.76	0.56-13.5		
**Perianal Disease**							0.6962
Absent	117	2	1.7				
Present	24	10	41.7	0.71	0.13-3.95		
**Fistulizing Disease**							0.9943
Absent	96	4	4.2				
Present	45	8	17.8	1.01	0.26-3.91		

Abbreviation: CI, confidence interval.

**Table 6.** Factors associated with medical therapy escalation.

	N	Escalation of Biologic Therapy		Logistic Regression			
		n	%	Odds Ratio	95% CI		*P*
					Lower	Upper	
**Multiple Surgeries**							0.5996
No	121	60	84.5				
Yes	20	11	15.5	0.76	0.27-2.11		
**Smoking History**							0.1434
Non smoker	52	20	28.2				
Smoker/Former smoker	89	51	71.8	1.83	0.82-4.09		
**Perianal Disease**							0.1990
Absent	117	60	84.5				
Present	24	11	15.5	2.21	0.62-0.29		
**Fistulizing Disease**							0.8118
Absent	96	50	70.4				
Present	45	21	29.6	0.91	0.42-0.81		

Abbreviation: CI, confidence interval.

## Discussion

This is an observational retrospective cohort study assessing the rate of endoscopic disease relapse and the incidence of long-term negative outcomes in operated CD patients stratified based on whether they were receiving biologic therapy at baseline colonoscopy. A total of 141 patients were included in our cohort, with a median follow-up of about 3 years. Notably, patients treated with biologic agents at baseline had a significantly lower rate of endoscopic recurrence (45.2% vs 80.2%, *P < .*0001) and histologic disease relapse (10.3% vs 0.0%, *P = .*0492) compared with those who were not. Additionally, a significant difference in the rate of long-term negative outcomes was also detected between the 2 study arms. Particularly, patients on biological therapy had a lower risk of hospitalization and surgery compared with those not treated (0.0% vs 12.1%, *P = .*0018). Similarly, the rate of medical therapy escalation was significantly lower in those who were treated at baseline (19.0% vs 62.6%, *P < .*0001). It is important to note that most individuals who at baseline were not receiving treatment initiated a biologic therapy during their disease course. This finding implies that most patients do require therapy. This is in line with the POCER study, which demonstrated that most patients do relapse later in their disease course.^16^ Our results have considerable relevance in daily clinical practice and have an important impact from an economic point of view. Growing evidence suggests that introduction of biologic therapy during early stages of CD with a top-down treatment strategy is associated with better clinical outcomes.^14,17^ Based on the results of our study, it is reasonable to speculate that operated patients could benefit from early treatment with biologic drugs as soon as they have passed the postoperative setting regardless of endoscopic disease recurrence or risk factors. Biologic therapy may prevent disease progression and improve the quality of life by protecting against hospitalization and further surgeries. In addition, it may also reduce the number of colonoscopies, which are costly, invasive, and poorly tolerated by patients.^18^ In Europe, the estimated direct medical costs per CD patient per year range from 2800€ to 6960€.^19^ Hospitalizations and surgeries account for approximately two-thirds of these costs.^19^ Such negative outcomes also have an impact on the individual’s productivity since they prevent patients from going to work.^20^ Treating patients with biological agents early could reduce the rate of long-term negative outcomes, leading to relevant direct and indirect cost savings.

To the best of our knowledge, this is the first study to evaluate whether postoperative treatment with biologics has an impact on the rate of endoscopic relapse and the incidence of long-term negative outcomes. Other strengths of this study include the long follow-up duration (approximately 3 years) and the adoption of a commonly accepted score to define endoscopic relapse in operated CD patients.^11,12^ Moreover, all patients underwent a colonoscopy at a specific time point (6-12 months) after surgery in line with current guidelines.^12^ However, some limitations also need to be addressed: it was a single-center study, and therefore the data need to be validated in other centers in order to confirm their reliability. Additionally, this study was conducted retrospectively, and histologic data were not available in all patients. However, histologic disease activity is not a treatment target in CD, and specimen collection is not regularly performed during colonoscopy, justifying the limited samples.^21^

Two main concerns limit the use of biologics in all operated patients: on the one hand, the fear of overtreatment exposing patients to the risk of adverse events; on the other hand, the mere economic aspect. However, accumulating evidence confirms the efficacy and safety of biologic agents in CD patients supporting their reliable use.^22–28^ Currently, adalimumab is the most widely used drug in this specific patient setting, but further studies are needed to evaluate the best therapeutic algorithm. To date, ustekinumab and vedolizumab are also available in the CD treatment armamentarium, which guarantee a reassuring safety profile.^3,26,27^ A randomized head-to-head trial compared adalimumab and ustekinumab for the management of moderate to severe CD patients, demonstrating that ustekinumab was not inferior to adalimumab.^28^ A dedicated study to evaluate the efficacy of ustekinumab in operated CD patients is warranted. Finally, the availability of biosimilars, the increasing use of drugs administered subcutaneously, and the imminent expiry of the ustekinumab and vedolizumab patents minimize the economic problem.^29–32^ It is important to mention that suboptimal control of inflammation in patients with CD increases the risk of negative outcomes that may require hospitalizations and surgery, resulting in a great economic burden which may surpass the cost of biologic agents.^14,33^

Our data should also be examined in the context of surgical studies like LIR!C and SUPREME-CD, showing that timing of surgery and type of surgery can reduce the risk of disease recurrence.^34,35^ Nevertheless, this risk is not canceled, as demonstrated by patients who relapse despite early surgery or KONO-S intervention, thus supporting the need for prophylactic therapy. However, the question concerning the duration of this treatment remains open. To date, there is little evidence regarding the EXIT strategies, and it is not known whether biological therapy can be discontinued without risk of recurrence. For this reason, we assume that therapy should be continued chronically.^36^ Discontinuation should be evaluated case-by-case only after a deep and lasting clinical, endoscopic, and radiological remission and after careful sharing with the patient of the risks and benefits of the therapeutic suspension.

A phase 4 randomized controlled study named “Prevention of Postoperative Endoscopic Recurrence With Endoscopy-driven Versus Systematic Biological Therapy (SOPRANO-CD; NCT05169593)” will enroll 292 operated CD patients in order to evaluate the rate of postoperative endoscopic recurrence and the need for unscheduled treatment adaptation. A group of patients will be treated with biologic agents immediately after surgery, and differently a second arm will receive endoscopy-driven induction of biologic therapy. The results of this study will be of great importance to define the best management of operated CD patients.

## Conclusion

Our results suggest that treatment with biologic agents may be beneficial in all patients postoperatively. Patients treated with biologic therapy had a decreased rate of endoscopic and histologic relapse. Moreover, biological therapy was associated with a reduced risk of long-term negative outcomes including surgery, hospitalization, and need for medical therapy escalation. In line with these data, early treatment with biological therapy postoperatively may prevent disease recurrence and consequently reduce the number of performed colonoscopies. Further prospective studies are necessary to confirm whether all operated CD patients should be treated with biologic agents postoperatively regardless of endoscopic activity and risk factors.

## Data Availability

The data underlying in this article will be shared on reasonable request to the corresponding author.
